# Chronic, Active Inflammation in Patients With Failed Total Knee Replacements Undergoing Revision Surgery

**DOI:** 10.1002/jor.24398

**Published:** 2019-07-23

**Authors:** Hannah L. Paish, Thomas Edward Baldock, Colin S. Gillespie, Alicia del Carpio Pons, Derek A. Mann, David J. Deehan, Lee A. Borthwick, Nicholas S. Kalson

**Affiliations:** ^1^ Fibrosis Research Group, Institute of Cellular Medicine Newcastle University Newcastle upon Tyne NE2 4HH United Kingdom; ^2^ Musculoskeletal Unit, Department of Orthopaedics, Freeman Hospital, Newcastle Hospitals NHS Trust Newcastle upon Tyne NE7 7DN United Kingdom; ^3^ School of Mathematics & Statistics Newcastle University Newcastle upon Tyne NE2 4HH United Kingdom

**Keywords:** fibrosis, inflammation, knee, arthroplasty, pain

## Abstract

Chronic pain and restricted knee motion is a significant problem following the total knee arthroplasty (TKA). The molecular pathogenesis of pain post‐TKA is not known and no targeted therapeutic intervention is available. The aim of this study was to investigate whether pro‐inflammatory mediators are elevated in revision knee patients, indicating an active, ongoing inflammatory process that may contribute to pain. Twelve key markers (pro‐inflammatory cytokines granulocyte‐macrophage colony‐stimulating factor [GM‐CSF], interleukin 5 [IL‐5], IL‐8 and IL‐10, chemokines CCL2, CCL3, CCL4, and CCL13, mediators of angiogenesis Flt‐1, vascular endothelial growth factor, and cell migration vascular cell adhesion molecule 1 and intercellular adhesion molecule 1) were measured in knee tissue and synovial fluid (SF) from primary TKA (*n* = 29) and revision patients (*n* = 32). Indications for surgery were osteoarthritis (OA) for primary TKA, and component loosening (*n* = 11), stiffness (*n* = 11), laxity pattern (*n* = 8), or progression of OA in patella resurfacing (*n* = 3) for revision surgery. Pain levels (WOMAC score) were higher in revision than primary patients (*p* ≤ 0.05). Time from primary to revision ranged from 8 months to 30 years (median 10 years). All markers were elevated in revision TKA; there was no trend toward decreasing levels with greater time from primary surgery for any marker studied in SF. Similar results were seen in knee tissue. We found no differences comparing indications for revision surgery (*p* ≥ 0.05). The elevation of inflammatory mediators in painful post‐TKA knees requiring revision suggests active, chronic inflammation. Characterization of upregulated markers provides rationale for targeted therapy, even many years from the primary surgery. © 2019 The Authors. *Journal of Orthopaedic Research* ^®^ published by Wiley Periodicals, Inc. on behalf of Orthopaedic Research Society. J Orthop Res 37:2316–2324, 2019

Primary total knee arthroplasty (TKA) is one of the most commonly undertaken elective orthopedic procedures, with >98,000 performed in 2016 in the United Kingdom.^1^ While the majority of patients have a successful outcome, with reduced pain and improved function,^2^ a significant proportion (10–20%) develop problems including joint stiffness, reduced range of knee motion and pain, which can ultimately require revision surgery.^3^

Extensive work has been undertaken to identify patient factors (e.g., male/female sex, co‐morbidities, mental health, and preoperative patient expectations)^4^ and surgical factors (e.g., implant design and surgical technique)^5^ that predict poor outcome following TKA. Increasingly, however, the role of patient biology as a potential driver of pain, stiffness and dissatisfaction is being considered.^6^

A relationship between levels of pro‐inflammatory mediators (interleukin 6 [IL‐6], IL‐8, CCL2, C‐reactive protein [CRP]) and pain levels in osteoarthritis has been well described.^7^, ^8^ However, our understanding of the role of these mediators in pain and stiffness following TKA is more limited. Gandhi et al.^9^ studied 28 patients undergoing TKA and found that higher preoperative levels of tumor necrosis factor α (TNF‐α), matrix metallopeptidase 13, and IL‐6 in synovial fluid (SF) were associated with reduced improvement in pain scores 2 years following TKA. Conversely, Zietek et al.^10^ measured TNF‐α levels in SF taken at time of primary surgery, and showed that higher levels of TNF‐α were positively correlated with improved pain scores at 6 weeks post‐surgery. Ugraş et al.^11^ measured SF levels of IL‐6 and CRP 24 h postoperatively (sampling through a drainage tube placed intra‐operatively) and found that higher levels of IL‐6 were associated with slower recovery. These studies demonstrate uncertainty currently surrounding the role of pro‐inflammatory cytokines in pain generation following TKA.

We and others have previously shown that the post‐TKA joint is associated with the persistence of myofibroblasts and extensive fibrotic remodeling.^12^, ^13^ More recently, we have investigated the expression of 39 soluble inflammatory proteins in SF, fat pad (FP), and synovial membrane (SM) from patients undergoing either primary or revision surgery.^14^ There was elevated expression of a significant number of these markers in each anatomical location: *n* = 26/39 markers in SF, *n* = 22/39 in infrapatellar FP and *n* = 10/39 in SM in revision versus primary knees.

The markers investigated in this study fulfilled two inclusion criteria; (i) they had previously been found significantly elevated in SF in revision TKR patients^14^; and (ii) they are well characterized as mediators of fibrosis, inflammation, and/or pain.^12^, ^13^, ^15^ Specifically we were interested in pro‐inflammatory and immune cell recruitment mediators, including the CCL family of chemokines. GM‐CSF is produced by fibroblasts, promotes neutrophil and macrophage proliferation and maturation, and is upregulated immediately postoperatively in serum following TKA.^16^ IL‐5 is a B‐cell activating cytokine elevated in serum immediately following total knee replacement surgery^17^ that drives fibrosis via Th2 B cell development and myofibroblast activation.^18^ IL‐8, a CXC chemokine involved in recruitment of neutrophils,^19^ has a positive correlation with IL‐1 expression, suggesting that fibroblasts exposed to IL‐1 in the surgical knee may be a source of IL‐8^14^. IL‐10 has been demonstrated to drive lung fibrosis when overexpressed, and this process is dependent on CCL2 signaling^20^ and is elevated in serum following knee surgery.^17^ CCL2 levels correlate positively with patient's pain levels following primary TKA, suggesting a role for CCL2 in pain pathogenesis, and recruitment of monocytes by knee fibroblasts in vitro is dependent on CCL2 signaling.^14^ CCL3 and CCL4 are elevated immediately following knee surgery^17^ and CCL13 is a key mediator of immune cell recruitment in chronic inflammation. Intercellular adhesion molecule 1 (ICAM‐1) is an adhesion molecule expressed by macrophages and other leukocytes and vascular cell adhesion molecule 1 (VCAM‐1) is expressed by activated endothelium and facilitates trans‐migration of immune cells into tissues. Soluble vascular endothelial growth factor (VEGF) and Flt‐1 are regulators of vessel growth; VEGF directly stimulates new vessel formation and growth of existing vessels, signaling through Flt‐1.^21^

However it is not known whether the inflammatory response post‐TKA is acute and resolving, or whether this process is chronic. To test this we examined the levels of key pro‐inflammatory mediators in SF and tissue of patients undergoing revision surgery and compared this to time elapsed from their primary TKA procedure.

Results presented here suggest that key, active pro‐inflammatory and matrix deposition markers are elevated in patients undergoing revision surgery, demonstrating the presence of active and ongoing chronic inflammation. These findings provide rationale for targeted biological therapy in knee pain and stiffness, even several years after the primary surgery. Furthermore, these key mediators could be molecular targets in patients with unexplained knee pain following TKR.

## METHODS

### Patient Recruitment and Ethics

This research has been conducted following ethical approval through the Newcastle Biobank (17/NE/0361).

### Tissue Collection and Patient Stratification

All patients undergoing revision surgery for failed primary TKA were included in the study over a two‐year period with infection as the only exclusion criterion. Level of evidence—III, retrospective cohort study comparing disease and control populations. SF, infrapatellar fat pad (FP), and synovial membrane (SM, suprapatellar pouch) were collected from patients undergoing either primary (*n* = 29) or revision (*n* = 33) TKA at the Freeman Hospital, Newcastle upon Tyne (Table 1). Patient reported outcome measures (Western Ontario & McMaster Universities Osteoarthritis Index, WOMAC scores) were recorded as routine practice in the Freeman hospital joint registry at the time of surgery. Revision TKA patients were stratified as those with osteolysis and loose components (*n* = 11), clinical diagnosis of fibrosis with loss of movement (*n* = 11), primary laxity pattern with functional instability (*n* = 8, see Supplementary Methods for details), and progression of osteoarthritis (previous patellofemoral resurfacing) (*n* = 3). The clinical definition of joint fibrosis and method for exclusion of infection was as previously described^12^, ^22^ (Supplementary Methods). Types of knee replacement in the revision cohort and surgical technique are described in Supplementary Methods. Histological analysis was performed on 9/33 patients (see Supplementary Methods).

**Table 1:** Patient Demographics

	Primary (*n* = 29)	All Revisions (*n* = 33)	*p* Value	Osteolysis, Loose Components	Primary Laxity Pattern (*n* = 8)	Progression of OA(PFJ Replacement, *n* = 3)	Clinical Diagnosisof Fibrosis (*n* = 11)
Age (years, mean, SEM)	66 ± 2	68 ± 2	0.76	70 ± 3.2	67 ± 5	69 ± 6	65 ± 4
Gender	17 M:12 F	16 M:17 F	0.88	5 M:6 F	4 M:4 F	3 F	7 M:4 F
BMI (Mean, SEM)	33 ± 1	33 ± 1	0.97	34 ± 2	33 ± 2	30 ± 2	34 ± 2
Time from primary to revision surgery (years, mean, SEM)	–	10 ± 1	–	16 ± 3	6 ± 1	6 ± 3	8 ± 2
WOMAC pain scores (Mean, SEM)	30 ± 3	52 ± 7	0.001	65 ± 5	49 ± 4	34 ± 7	38 ± 4

No difference was found comparing age, gender, or body mass index (BMI) in revision versus primary patients, or comparing groups according to indication for revision (*p* = > 0.05, *U* test). Pain was significantly higher in revision versus primary patients (*p* = < 0.001).

SEM, standard error of mean.

### Infrapatellar Fat Pad and Synovial Membrane

Tissue was stored at 4°C in tissue culture media containing 10% fetal calf serum, 1% l‐glutamine, 100 units/ml penicillin, and 100 μg/ml streptomycin before processing. Tissue was homogenized in radioimmunoprecipitation assay buffer supplemented with protease and phosphatase inhibitors using a bead homogenizer (TissueLyserII; Qiagen, Germantown, MD). Homogenized samples were normalized to 1 mg/ml total protein (protein concentration measured using a BCA protein assay (Pierce) as per manufacturer's instructions) and stored at −80°C before analysis.

### Multi‐Array Protein Assays

Not all patients had fluid and both tissue samples collected at the time of surgery. SF (primary *n* = 21 patients, revision *n* = 24 patients), and tissue homogenates from infrapatellar FP (primary *n* = 28 patients, revision *n* = 32 patients) and SM (primary *n* = 29 patients, revision *n* = 32 patients) were assessed for expression of 12 protein markers using human V‐Plex electrochemiluminescence detection kits from Meso‐Scale Discovery as per manufacturer's instructions. Analysis of results was performed using the MSD Discovery Workbench analysis software.

### Statistical Analysis

Differences in patient demographics were examined using Mann–Whitney *U* (Graph Pad, Prism, San Diego, CA, version 7). Time from primary TKA to revision was modeled using marker levels, age, and body mass index (BMI) using multiple linear regression (performed in R, available at http://www.r-project.org). Differences in marker levels between primary and revision patients, and between different revision groups were examined using Mann–Whitney *U*. Data are presented as mean ± standard error of the mean (SEM). *p* < 0.05 were regarded as significant.

## RESULTS

### Pain Level in Revision and Primary TKA Patients

There were no differences in age, BMI, and sex between the primary and revision groups, and within the revision group when comparing different indications for revision surgery (Table 1). At time of surgery, patient reported pain scores (WOMAC) were higher in revision than primary TKA patients (52 ± 7 vs. 30 ± 3, *p* = 0.001, Table 1). No differences in pain scores within the revision group when comparing different indications for revision surgery were observed (*p* = 0.13).

### Elevation of Key Pro‐Inflammatory Cytokines, Chemokines and Mediators of Fibrosis Many Years After Primary Surgery

In total 12 markers were selected, all of which were increased in SF when comparing revision to primary patients (at a significance threshold of *p* = < 0.01 level, *U* test, Table 2, 14). In addition to quantifying these markers in SF, we also examined their levels in FP (Supplementary Results, Table S3) and SM tissue (Supplementary Results, Table S4). In FP tissue *n* = 10/12 markers were significantly elevated in revision patients compared to primary and in SM *n* = 4/12 markers were significantly elevated compared with primary.

**Table 2:** Synovial Fluid

Marker	Primary (*n* = 21)Mean ± SEM (pg/ml)	All Revisions (*n* = 24)Mean ± SEM (pg/ml)	Primary vs. Revision TKA		Osteolysis, Loose Components (*n* = 11)Mean ± SEM (pg/ml)	Primary Laxity Pattern (*n* = 7)Mean ± SEM (pg/ml)	Progression of OA (PFJ Replacement, *n* = 1)Mean ± SEM (pg/ml)	Clinical Diagnosis of Fibrosis (*n* = 7)Mean ± SEM (pg/ml)
			*p* Value	Significance				
GM‐CSF	0.5 ± 0.2	1.3 ± 0.3	<0.01	^**^	1.8 ± 0.5	0.5 ± 0.2	0	1.6 ± 0.5
IL‐5	0.4 ± 0.1	2.2 ± 0.8	<0.001	^***^	1.6 ± 0.4	4.1 ± 2.7	0	1.2 ± 0.4
IL‐8	80 ± 31	3,147 ± 1,552	<0.0001	^****^	6,296 ± 3,996	777 ± 215	61	1,907 ± 829
IL‐10	0.8 ± 0.5	1.6 ± 0.4	<0.0001	^****^	2.6 ± 0.9	0.8 ± 0.2	0	1.2 ± 0.6
CCL2	417 ± 38	5,285 ± 1,004	<0.0001	^****^	5,902 ± 1,233	3233 ± 791	318	7,254 ± 2,824
CCL3	15.6 ± 3.3	177 ± 51	<0.0001	^****^	285 ± 125	96 ± 16	16	142 ± 54
CCL4	69.6 ± 8	258 ± 54	<0.0001	^****^	368 ± ± 131	213 ± 39	36	192 ± 51
CCL13	42.5 ± 20	220 ± 76	<0.0001	^****^	211 ± 92	67 ± 22	14	211 ± 92
Flt‐1	97.6 ± 12	1575 ± 612	<0.0001	^****^	3,025 ± 1,512	391 ± 170	33	1,116 ± 443
VEGF	396.1 ± 46	1724 ± 268	<0.0001	^****^	1,589 ± 220	1,081 ± 292	133	2,768 ± 680
VCAM‐1	105,613 ± 7,290	134,770 ± 7,434	<0.05	^*^	138,249 ± 10,066	151,817 ± 15,596	96,090	118,775 ± 13,418
ICAM‐1	862,890 ± 12,439	150,915 ± 24118	<0.05	^*^	189,061 ± 39,854	168,010 ± 58,990	31,383	101,851 ± 18,441

Levels of markers in synovial fluid. Comparing revision vs. primary synovial fluid all markers were significantly upregulated (*p* ≤ 0.05, *U* test). No significant differences were seen comparing different indications for revision surgery (*p* ≥ 0.05).

GM‐CSF, granulocyte‐macrophage colony‐stimulating factor; ICAM‐1, intercellular adhesion molecule 1; IL, interleukin; VCAM‐1, vascular cell adhesion molecule 1; VEGF, vascular endothelial growth factor.

^*^ *p* < 0.05.

^**^ *p* < 0.01.

^***^ *p* < 0.001.

^****^ *p* < 0.0001.

We next asked whether the levels of markers found to be elevated in post‐TKA knees correlated with time from primary surgery. To do this we fitted a multiple linear regression model while controlling for age and BMI.

**Figure 1:**
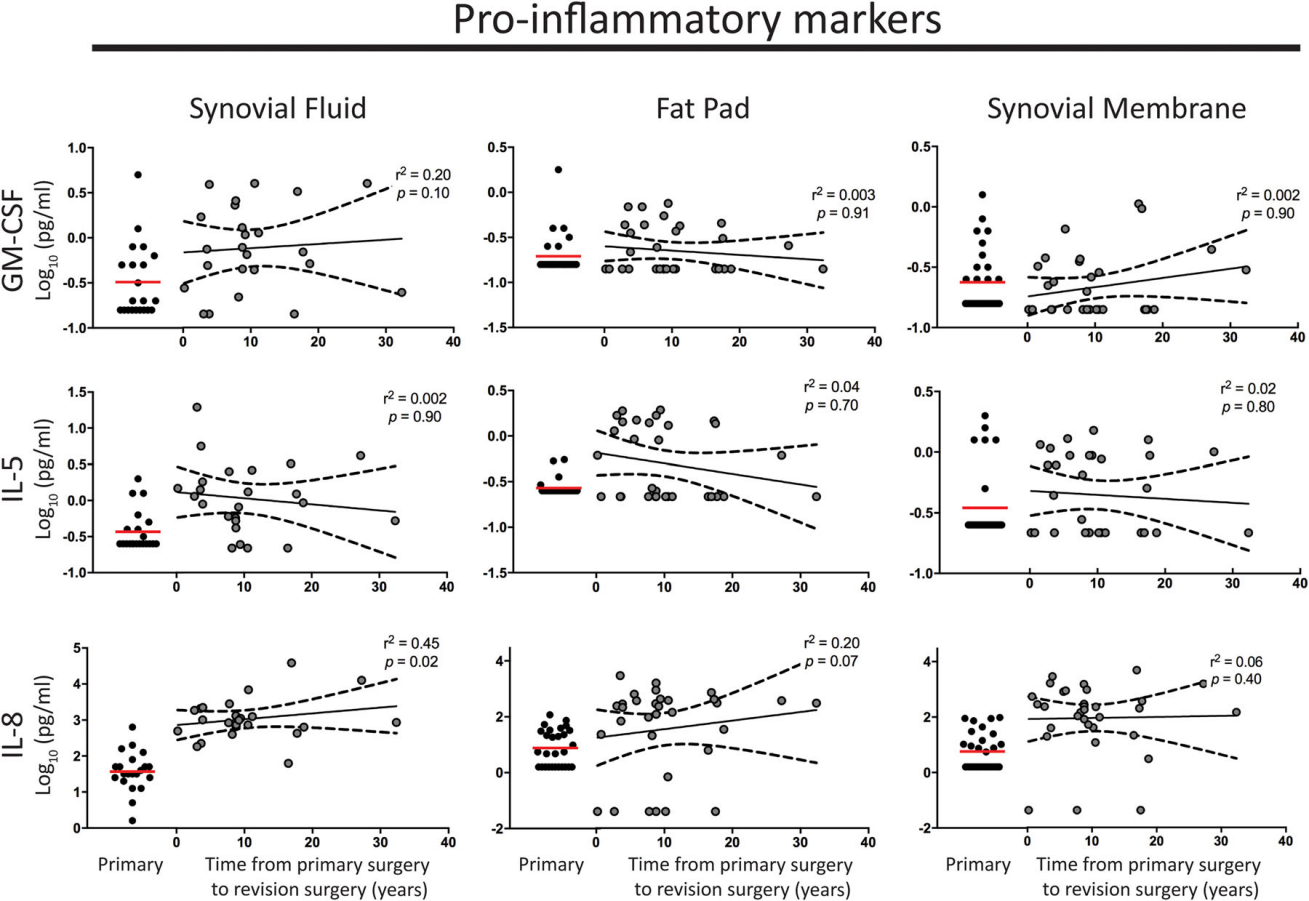
Elevated levels of pro‐inflammatory mediators granulocyte‐macrophage colony‐stimulating factor (GM‐CSF), interleukin 5 (IL‐5), and IL‐8 are maintained over time following primary total knee arthroplasty (TKA) in synovial fluid and tissue in patients with joint fibrosis. No significant change in the levels of pro‐inflammatory mediators GM‐CSF, IL‐5, and IL‐8 was found over time from primary TKA surgery in synovial fluid, fat pad, or synovial membrane tissue. The red line indicates the mean level in primary tissue for comparison. The black line represents the mean and black long dashed lines the 95% confidence interval. A linear regression model was used; *r* ^2^ and *p* values are presented for each analysis. [Color figure can be viewed at wileyonlinelibrary.com]

Inflammation, fibrosis, and pain are dependent on immune cell recruitment and activation. We have previously found infiltration of CD68 + monocytes into fibrotic tissue.^14^ Therefore, we first examined GM‐CSF (increased ~threefold in SF of revision versus primary knees), IL‐5 (increased sixfold), IL‐8 (increased 39‐fold), and IL‐10 (increased twofold). All four markers were elevated even many years after surgery in SF, FP, and SM (Fig. 1). Seven patients had GM‐CSF levels >10‐fold higher than in primary TKA patients >10 years after their initial surgery.

Elevation of IL‐6 and of tumor necrosis factor‐α (TNF‐α) at time of surgery in SF has previously been shown to predict worse pain improvement 2 years post‐TKA.^9^ However, IL‐6 was not detected in SF in revision or primary samples in this study. Although TNF‐α was >3‐fold increased in SF (*p* ≤ 0.0001) and was detectable in all SF samples, TNF‐α was not robustly detected in FP (0/28 primary, 9/32 revision) or SM tissue (2/28 primary, 9/32 revision).

**Figure 2:**
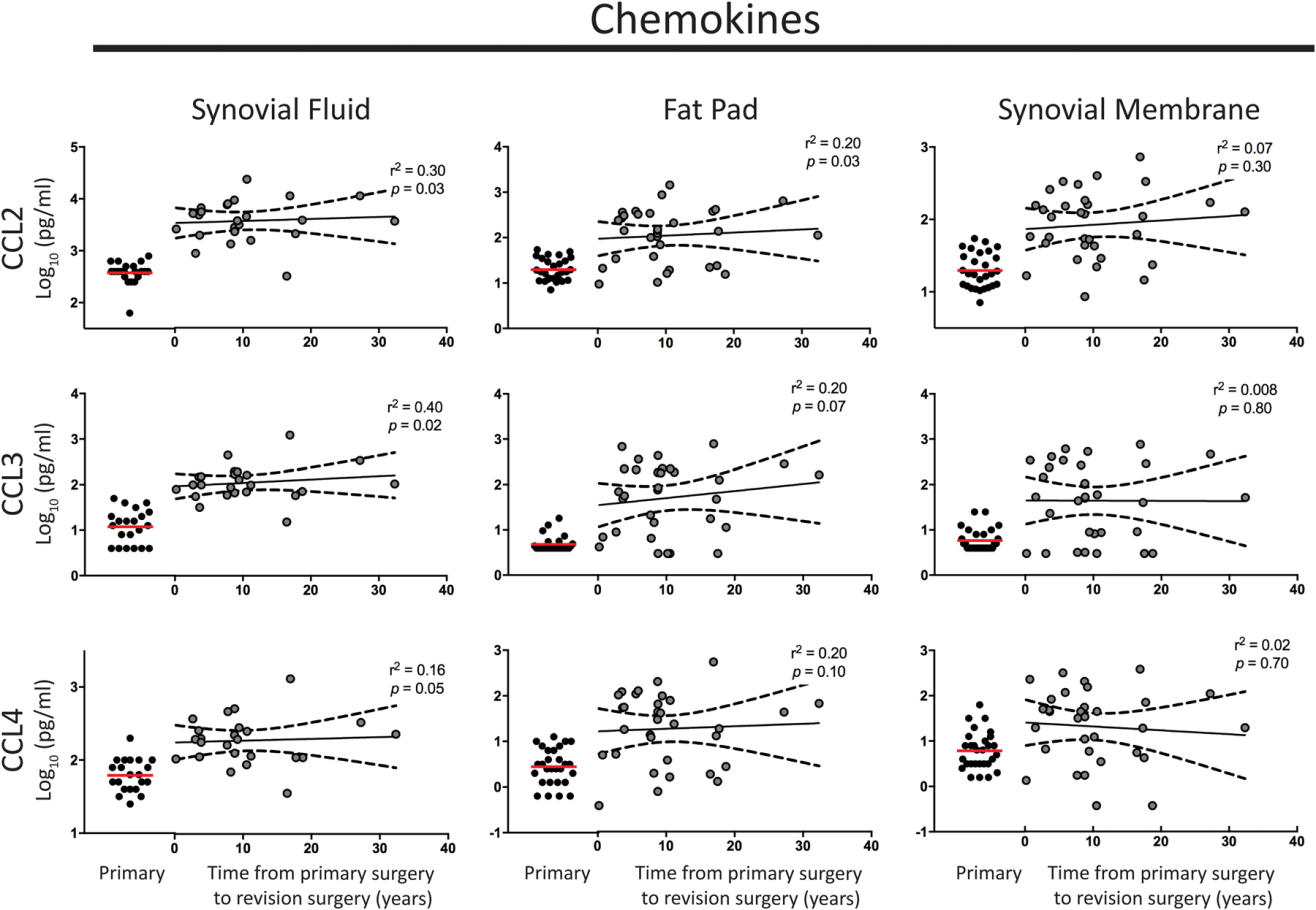
Elevated levels of Immune cell recruitment mediators CCL2, CCL3, and CCL4 in synovial fluid and tissue following primary total knee arthroplasty (TKA) in patients with joint fibrosis. No significant change in the levels of CCL2, CCL3, and CCL4 was found over time from primary TKA surgery in synovial fluid, fat pad, or synovial membrane tissue. The red line indicates the mean level in primary tissue for comparison. The black line represents the mean and black long dashed lines the 95% confidence interval. A linear regression model was used; *r* ^2^ and *p* values are presented for each analysis. [Color figure can be viewed at wileyonlinelibrary.com]

The relationship between IL‐10 and CCL2 in driving chronic fibrosis^20^ led us to investigate the temporal expression of CCL2 (increased 13‐fold in SF revision vs. primary), and other CCL family members (CCL3, 4, and 13, increased >5‐fold in SF). All four CCLs were elevated in fluid and tissue even many years after primary surgery (Fig. 2).

**Figure 3:**
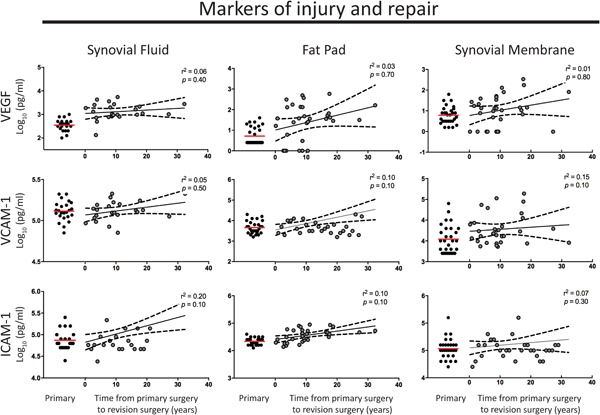
Elevated levels of vascular endothelial growth factor (VEGF), vascular cell adhesion molecule 1 (VCAM‐1), and Intercellular adhesion molecule 1 (ICAM‐1) in synovial fluid and tissue following primary total knee arthroplasty (TKA) in patients with joint fibrosis. No significant change in the levels of VEGF, VCAM‐1, and ICAM‐1 was found over time from primary TKA surgery in synovial fluid, fat pad, or synovial membrane tissue. The red line indicates the mean level in primary tissue for comparison. The black line represents the mean and black long dashed lines the 95% confidence interval. A linear regression model was used; *r* ^2^ and *p* values are presented for each analysis. [Color figure can be viewed at wileyonlinelibrary.com]

Having demonstrated chronic elevation of pro‐inflammatory and immune cell recruitment mediators, we asked whether cell adhesion/migration markers (ICAM‐1, VCAM‐1) and proangiogenic mediators (VEGF and Flt‐1) also remain elevated. ICAM‐1 was upregulated twofold in SF and FP (*p* ≤ 0.05, Table 2). Both VEGF and Flt‐1 are upregulated in revision SF (fourfold and 16‐fold). Again, there was no reduction in level of these four markers with greater time from surgery in both fluid and tissue (Fig. 3).

No marker studied demonstrated a reduction in level with greater time from surgery (Figs. 1–3, tested in a linear regression model). This finding suggests a chronic type‐2 inflammatory response in revision patients.

### No Significant Differences in Inflammatory Markers Between Different Revision Groups

**Figure 4:**
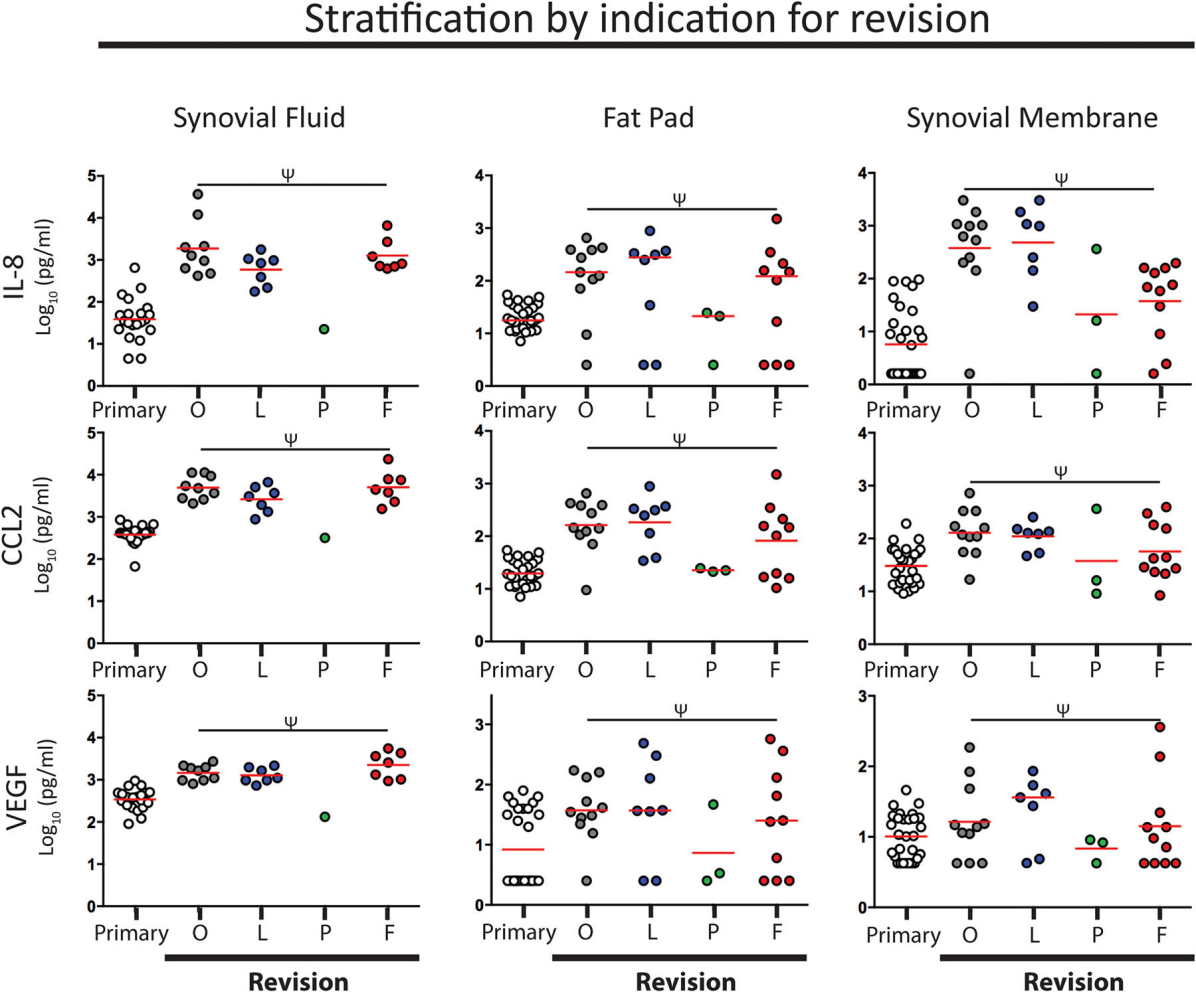
No differences in inflammatory molecule profile between different indications for revision. Analysis comparing four different revision groups failed to demonstrate any significant differences in any of the molecular mediators investigated. Interleukin 8 (IL‐8), CCL2, and vascular endothelial growth factor (VEGF) are shown as examples. The mean is shown for each group as a red line. Ψ denotes *p* ≥ 0.05. Indications for revision were osteolyisis (O), laxity (L), revision of patello‐femoral replacement (P), and fibrosis (F). [Color figure can be viewed at wileyonlinelibrary.com]

As we had a heterogeneous group of revision patients who were undergoing surgery for one of four different indications we examined possible differences in levels of inflammatory markers between different revision groups. We found no significant differences in any of the markers studied in SF, FP, or SM tissue (*p* ≥ 0.05 for all tests; example graphs are shown in Figure 4, data is presented in Tables 2, S3 and S4 [Supplementary Results]).

## DISCUSSION

Knee pain and stiffness is a devastating complication following total knee replacement yet our understanding of the biology of the painful TKA remains incomplete.^6^ Previous studies have tested the hypothesis that the level of inflammation pre‐ or peri‐operatively is correlated with clinical outcome and/or pain.^10^, ^11^ Here, we have studied a group of patients undergoing revision surgery for all causes (having excluded infection). We report that in patients undergoing revision surgery, who have higher pain scores than primary TKA patients, there is elevation of key inflammatory mediators in SF and knee tissue. This is present irrespective of time from primary surgery, with low *r* ^2^ and *p* values in multiple linear regression for all markers tested against time. These findings suggest that targeted modulation of chronic inflammation may offer therapeutic benefit in the treatment of pain following TKA.

Stiffness and joint fibrosis is a significant problem following TKA. We have previously shown that fibrotic, dense collagenous scar tissue is found in all patients undergoing aseptic revision surgery for failed primary TKA.^12^ The fibrotic tissue is characterized by the deposition of a dense, disorganized extracellular matrix of collagen^23^ populated by myofibroblasts.^12^, ^13^ Although all revision knees in this cohort have joint fibrosis, presumably there are differences in the anatomic location and amount of fibrotic tissue present. We have not been able to determine whether fibrosis is a result of an ongoing inflammatory state caused by a failing primary TKA, or is normal scar tissue found post‐TKA. Consequently differences in the molecular biology of the stiff TKA must be further investigated and dissected out from those seen in TKAs that have failed for other reasons (e.g., loosening, component mal‐position). Animal models of joint fibrosis have been developed and may provide further mechanistic insights into the disease pathogenesis.^24^ Histological analysis in this study showed polyethylene wear particles that could have a pathogenic role in driving chronic inflammation.

Treatment of joint fibrosis is currently limited to physical therapy. Patients resistant to nonoperative treatment require arthroscopic or more invasive surgical procedures to excise and remove the soft tissue contractures.^25^ The outcomes of surgically treated posttraumatic fibrosis of the knee are poor, with most patients unable to return to pre‐injury level of function.^26^, ^27^ Our understanding of the molecular pathogenesis of joint fibrosis is incomplete and consequently no targeted therapy is available. IL‐1 receptor is expressed in high levels on the surface of knee fibroblasts,^15^ and one pilot case series reported improved range of motion following intra‐articular injection of Anakinra, an IL‐1 receptor antagonist,^28^ demonstrating the potential for targeted therapy. Development of fibrosis and consequently knee stiffness, manifest as restricted range of motion (ROM), is associated with increased pain postoperatively,^29^ suggesting that chronic inflammation may be a common pathway for development of pain and restricted ROM due to excessive scarring.

Suppressing the inflammatory response peri‐operatively has been investigated in a randomized trial of TKA patients.^30^ Administration of three doses of intravenous hydrocortisone decreased systemic inflammation (measured by serum IL‐6 levels) 24 h postoperatively. Patients receiving steroid had improved ROM at time of discharge, and although no long‐term follow‐up data is available, dampening inflammation may provide a potential clinical benefit.

Previous studies have measured pre‐ or peri‐operative levels of TNF‐α 9, 10 and IL‐6.^9^, ^11^ We found TNF‐α threefold higher in revision versus primary SF, supporting a possible role in promoting pain and inflammation. However, IL‐6 was not upregulated in revision knees suggesting that IL‐6 is most useful as an early pre‐/peri‐operative biomarker.

In this cohort pain scores were higher in failed TKA patients than patients undergoing primary surgery for osteoarthritis. This demonstrates the challenges faced to achieve a satisfactory outcome following revision, and is consistent with previously reported higher levels of chronic pain after revision TKA compared with following primary TKA.^31^

Several limitations to the present study should be noted. Revision patients studied here were a heterogeneous group undergoing surgery for all causes. We have not been able to robustly identify differences in the molecular profile comparing different indications for revision. There is no previously published data on differences in inflammatory microenvironment of the post‐TKA knee to allow a power calculation to be performed. Revision surgery is relatively infrequently required following TKA, and despite an unbiased patient selection strategy for our revision cohort to maximize the number of patients recruited to the study, with the only rule‐out clinical condition being infection, we are likely to be underpowered to detect differences between different indications for revision surgery in this exploratory experimental study. We have compared revision patients to primary patients with advanced osteoarthritis; the ideal control group would be TKA patients with a well‐functioning prosthesis and this will be tackled in future studies. Biomarker changes may be age‐related, and although we controlled for age in multiple linear regression analysis we cannot exclude an effect of age in revision patients, particularly elderly patients who underwent primary surgery >20 years ago. We were unable to control for different analgesic regimens between patients, which could influence outcomes and were not able to differentiate neuropathic pain, which may have a different pathogenesis and is known to occur in a sub‐set of TKA patients.^32^

Data presented in this report suggest that the levels of key biologically active pro‐inflammatory mediators are locally elevated at time of revision surgery many years after the initial procedure in failed TKAs. The painful,^14^ fibrotic^12^ post‐TKA joint acquires a state of unresolved inflammation with a signature resembling that of a chronic allergic reaction.^33^ Markers with this temporal regulation include those involved in matrix formation (VEGF, Flt‐1) and the inflammatory cytokines GM‐CSF, IL‐5 and IL‐8, and cell recruitment chemokines CCL2, CCL3 and CCL4. Similar results were obtained in tissue and SF. This finding has implications when considering therapeutic intervention. The elevation of these markers indicates an active disease process, thus offering the possibility to interrupt activated molecular pathways. Far from being quiescent, the knee exists in state of chronic inflammation. In addition to reduced ROM knee fibrosis is a painful condition.^29^, ^34^ CCL2 correlates positively with pain levels^14^; blocking CCL2 signaling could reduce pain symptoms in these patients.

Although we report elevation of inflammatory mediators in revision patients many years from primary surgery, the question remains as to whether these mediators are important in pathogenesis of TKA failure. Longitudinal sampling is required to determine the contribution of these mediators to the pathogenesis of disease (e.g., fibrosis) that led to requirement of revision surgery. In addition, these mediators may be important in the 10–20% of patients that report unexplained knee pain and dissatisfaction following TKA.^35^ A longitudinal, prospective study is required to determine whether chronic inflammation is present in patients with unexplained pain not requiring revision surgery.

A number of biologically active markers involved in inflammation and matrix deposition were significantly elevated many years following primary TKA. Approved therapeutics are available that target these molecules, for example, CCL2, IL‐8, and CCL3, and further studies are required to evaluate these compounds in patients with stiff, painful failed TKAs. In addition, prospective examination of the painful, stiff TKR is now required to determine (i) whether chronic inflammation is present in patients without a specific cause for their pain, and (ii) to identify molecular mediators to direct targeted immunomodulatory adjunctive therapy.

## ACKNOWLEDGMENTS

Supported by grants from the Medical Research Council (MRC) (MR/K001949/1 and MR/R023026/1), Wellcome Trust (204787/Z/16/Z), Newcastle Surgical Training Center, The Royal College of Surgeons of Edinburgh and the National Institute for Health Research (NIHR) Newcastle Biomedical Research Center, based at Newcastle upon Tyne Hospitals NHS Foundation Trust and Newcastle University. The views expressed are those of the author(s) and not necessarily those of the NHS, the NIHR, or the Department of Health.

## Supporting information

Supporting information

Supporting information

## References

[1] National Joint Registry Editorial Board . National Joint Registry Annual Report 2017. Available at: https://www.njrreports.org.uk.

[2] Skou ST , Roos EM , Laursen MB , et al. 2015 A randomized, controlled trial of total knee replacement. N Engl J Med 373:1597–1606. 26488691 10.1056/NEJMoa1505467

[3] Sharkey PF , Lichstein PM , Shen C , et al. 2014 Why are total knee arthroplasties failing today‐has anything changed after 10 years? J Arthroplasty 29:1774–1778. 25007726 10.1016/j.arth.2013.07.024

[4] Barlow T , Clark T , Dunbar M , et al. 2016 The effect of expectation on satisfaction in total knee replacements: a systematic review. Springerplus 5:167. 27026864 10.1186/s40064-016-1804-6 PMC4766134

[5] Ahmed I , Paraoan V , Bhatt D , et al. 2018 Tibial component sizing and alignment of TKR components does not significantly affect patient reported outcome measures at six months. A case series of 474 participants. Int J Surg 52:67–73. 29471154 10.1016/j.ijsu.2018.02.039

[6] Preston S , Petrera M , Kim C , et al. 2016 Towards an understanding of the painful total knee: what is the role of patient biology? Curr Rev Musculoskelet Med 9:388–395. 27613710 10.1007/s12178-016-9363-6 PMC5127943

[7] Stürmer T , Brenner H , Koenig W , Günther K‐P . 2004 Severity and extent of osteoarthritis and low grade systemic inflammation as assessed by high sensitivity C reactive protein. Ann Rheum Dis 63:200–205. 14722211 10.1136/ard.2003.007674 PMC1754873

[8] Monibi F , Roller BL , Stoker A , et al. 2015 Identification of synovial fluid biomarkers for knee osteoarthritis and correlation with radiographic assessment. J Knee Surg, 29:242–247. 25927354 10.1055/s-0035-1549022

[9] Gandhi R , Santone D , Takahashi M , et al. 2013 Inflammatory predictors of ongoing pain 2 years following knee replacement surgery. Knee 20:316–318. 23157967 10.1016/j.knee.2012.10.015

[10] Zietek P , Dziedziejko V , Safranow K , et al. 2016 TNF‐α concentrations in pre‐operative synovial fluid for predicting early post‐operative function and pain after fast‐track total knee arthroplasty. Knee 23:1044–1048. 27634599 10.1016/j.knee.2016.02.013

[11] Ugraş AA , Kural C , Kural A , et al. 2011 Which is more important after total knee arthroplasty: Local inflammatory response or systemic inflammatory response? The Knee 18:113–116. 20466551 10.1016/j.knee.2010.03.004

[12] Abdul N , Dixon D , Walker A , et al. 2015 Fibrosis is a common outcome following total knee arthroplasty. Sci Rep 5:16469. 26553967 10.1038/srep16469 PMC4639721

[13] Unterhauser FN , Bosch U , Zeichen J , Weiler A . 2004 Alpha‐smooth muscle actin containing contractile fibroblastic cells in human knee arthrofibrosis tissue Winner of the AGA‐DonJoy Award 2003. Arch Orthop Trauma Surg 124:585–591. 15378321 10.1007/s00402-004-0742-x

[14] Paish HL , Kalson NS , Smith GR , et al. 2017 Fibroblasts promote inflammation and pain via interleukin‐1α‐induction of the monocyte chemoattractant CCL2. Am J Pathol 188:696–714. 29248462 10.1016/j.ajpath.2017.11.007 PMC5842035

[15] Dixon D , Coates J , Del Carpio Pons A , et al. 2015 A potential mode of action for Anakinra in patients with arthrofibrosis following total knee arthroplasty. Sci Rep 5:16466. 26553966 10.1038/srep16466 PMC4639732

[16] Henriksen VT , Rogers VE , Rasmussen GL , et al. 2014 Pro‐inflammatory cytokines mediate the decrease in serum 25(OH)D concentrations after total knee arthroplasty? Med Hypotheses 82:134–137. 24332533 10.1016/j.mehy.2013.11.020

[17] Hirsch J , Vacas S , Terrando N , et al. 2016 Perioperative cerebrospinal fluid and plasma inflammatory markers after orthopedic surgery. J Neuroinflammation 13:211. 27577265 10.1186/s12974-016-0681-9 PMC5006595

[18] Gieseck RL , Wilson MS , Wynn TA . 2018 Type 2 immunity in tissue repair and fibrosis. Nat Rev Immunol 18:62–76. 28853443 10.1038/nri.2017.90

[19] Borthwick LA . 2016 The IL‐1 cytokine family and its role in inflammation and fibrosis in the lung. Semin Immunopathol 38:1–18. 27001429 10.1007/s00281-016-0559-z PMC4896974

[20] Sun L , Louie MC , Vannella KM , et al. 2011 New concepts of IL‐10‐induced lung fibrosis: fibrocyte recruitment and M2 activation in a CCL2/CCR2 axis. Am J Physiol Lung Cell Mol Physiol 300:L341–L353. 21131395 10.1152/ajplung.00122.2010 PMC3064283

[21] Failla CM , Carbo M , Morea V . 2018 Positive and negative regulation of angiogenesis by soluble vascular endothelial growth factor receptor‐1. Int J Mol Sci 19:1306. 10.3390/ijms19051306 PMC5983705 29702562

[22] Kalson NS , Deehan DJ , Mann DA , Borthwick LA . 2016 International consensus definition of post‐surgical knee joint fibrosis. Bone Joint J, 98‐B:1479–1488. 10.1302/0301-620X.98B10.37957 27803223

[23] Freeman TA , Parvizi J , Valle Dela CJ , Steinbeck MJ . 2010 Mast cells and hypoxia drive tissue metaplasia and heterotopic ossification in idiopathic arthrofibrosis after total knee arthroplasty. Fibrogenesis Tissue Repair 3:17. 20809936 10.1186/1755-1536-3-17 PMC2940819

[24] Barlow JD , Morrey ME , Hartzler RU , et al. 2016 Effectiveness of rosiglitazone in reducing flexion contracture in a rabbit model of arthrofibrosis with surgical capsular release: a biomechanical, histological, and genetic analysis. Bone Joint Res 5:11–17. 26813567 10.1302/2046-3758.51.2000593 PMC5009236

[25] Magit D , Wolff A , Sutton K , Medvecky MJ . 2007 Arthrofibrosis of the knee. J Am Acad Orthop Surg 15:682–694. 17989419 10.5435/00124635-200711000-00007

[26] Wang J‐H , Zhao J‐Z , He Y‐H . 2006 A new treatment strategy for severe arthrofibrosis of the knee. A review of twenty‐two cases. J Bone Joint Surg Am 88:1245–1250. 16757757 10.2106/JBJS.E.00646

[27] Millett PJ , Williams RJ , Wickiewicz TL . 1999 Open debridement and soft tissue release as a salvage procedure for the severely arthrofibrotic knee. Am J Sports Med 27:552–561. 10496569 10.1177/03635465990270050201

[28] Brown CA , Toth AP , Magnussen B . 2010 Clinical benefits of intra‐articular anakinra for arthrofibrosis. Orthopedics 33:877. 21162510 10.3928/01477447-20101021-09

[29] Mayr HO , Weig TG , Plitz W . 2004 Arthrofibrosis following ACL reconstruction—reasons and outcome. Arch Orthop Trauma Surg 124:518–522. 15480713 10.1007/s00402-004-0718-x

[30] Jules‐Elysee KM , Wilfred SE , Memtsoudis SG , et al. 2012 Steroid modulation of cytokine release and desmosine levels in bilateral total knee replacement: a prospective, double‐blind, randomized controlled trial. JBJS 94:2120–2127. 10.2106/JBJS.K.00995 23097096

[31] Petersen KK , Simonsen O , Laursen MB , et al. 2015 Chronic postoperative pain after primary and revision total knee arthroplasty. Clin J Pain 31:1–6. 25485953 10.1097/AJP.0000000000000146

[32] Phillips JRA , Hopwood B , Arthur C , et al. 2014 The natural history of pain and neuropathic pain after knee replacement: a prospective cohort study of the point prevalence of pain and neuropathic pain to a minimum three‐year follow‐up. Bone Joint J 9:1227–1233. 10.1302/0301-620X.96B9.33756 25183595

[33] Galli SJ , Tsai M , Piliponsky AM . 2008 The development of allergic inflammation. Nature 454:445–454. 18650915 10.1038/nature07204 PMC3573758

[34] Lavernia C , Cardona D , Rossi MD , Lee D . 2008 Multimodal pain management and arthrofibrosis. J Arthroplasty 23:74–79. 10.1016/j.arth.2008.03.012 18722306

[35] Becker R , Bonnin M , Hofmann S . 2011 The painful knee after total knee arthroplasty. Knee Surg Sports Traumatol Arthrosc 19:1409–1410. 21800166 10.1007/s00167-011-1625-7

