# Supplementary material for: Chronic, Active Inflammation in Patients With Failed Total Knee Replacements Undergoing Revision Surgery
Source: J Orthop Res. 2019 Jul 23;37(11):2316–24. doi: 10.1002/jor.24398 (PMC6851711; doi:10.1002/jor.24398)
Supplement: Supplementary file 1 — Supporting information [file JOR-37-2316-s001.docx]

**Supplementary Methods**

**Definition of joint fibrosis and exclusion of infection:** For the purposes of our study we used a definition of primary joint fibrosis as previously described ^13,14^. To exclude infection pre-operative workup included CRP (all patients) and joint aspiration (7/33 revision patients). Joint aspiration was carried out when the treating clinician felt that infection needed to be excluded. CRP was <30 in all revision patients and all pre-operative joint aspiration samples were negative following extended culture. Infection was definitively ruled out in all revision patients by microbiological analysis of synovial fluid and of multiple tissue samples taken at time of revision surgery. All revision patients had negative extended culture of both intra-operative synovial fluid and tissue samples. A minimum of three and a maximum of six tissue samples were taken intra-operatively according to prosthetic joint infection guidelines^26^. As is standard practice all orthopaedic theatre samples underwent extended enrichment culture for 10 days. However, we cannot rule out a role for organisms such as Propionibacterium acnes, which are challenging to culture, and may be an under-recognised cause of knee prosthetic joint infection ^33,34^.

**Histological Analysis of Tissue Samples.** Formal histology reports were available for 9/33 revision patients. All reports state that dense, hypocellular, heavily collagenised fibrous tissue foreign body giant cell containing small transparent, strongly birefringent foreign particles were identified. The appearances are those of extensive fibrosis together with a focal giant cell reaction to foreign material, suggestive of Polyethylene, in keeping with previous surgery.

**Type of knee replacement in revision cohort:** In the revision cohort the primary knee replacements were: 16 PFC (Sigma), 6 Kinemax (Howmedica), 2 Triathlon (Stryker), 1 genesis II (Smith and Nephew), 1 Oxford Uni-compartmental (Zimmer), 1 Vanguard (Biomet), 1 AGC (Biomet), 1 IB2 (Zimmer), 1 Noiles (DePuy) and 3 primary patella-femoral resurfacing. Six revision patients with a total knee replacement *in situ* also had a patella-femoral resurfacing.

**Reason for revision surgery - instability:** Eight patients were revised for instability; 6/8 had lost PCL required revision to a higher constraint prosthesis and 2/8 had mid-point laxity and the collateral ligaments were lax in full extension and underwent revision to a higher constraint implant. Eleven were revised for osteolysis and loose components, three for progression of osteoarthritis (patellofemoral resurfacing) and 11 with a clinical diagnosis of fibrosis with loss of movement (n=11) ^14^.

**Surgical Technique:** Routine practice in our unit includes excision of the infra-patella fat pad. All primary patients in the study had their IFP removed. In the revision cohort, 24 had primary surgery performed in our unit and therefore had their IFP removed in the primary procedure. The remaining nine revision patients had surgery elsewhere and we do now know whether the IFP was removed in their primary procedure. The infra-patellar fat pad tissue resected in revision surgery was from an anatomically matched site to the normal fat pad tissue found in the primary knee and resected in primary replacement surgery.
