# Supplementary material for: Chronic, Active Inflammation in Patients With Failed Total Knee Replacements Undergoing Revision Surgery
Source: J Orthop Res. 2019 Jul 23;37(11):2316–24. doi: 10.1002/jor.24398 (PMC6851711; doi:10.1002/jor.24398)
Supplement: Supplementary file 2 — Supporting information [file JOR-37-2316-s002.docx]

| Marker | Primary (n=28) | All revisions (n=32) | Primary versus Revision TKA | | Osteolysis, loose components (n=11) | Primary laxity pattern (n=8) | Progression of OA  (PFJ replacement, n=3) | Clinical diagnosis  of fibrosis (n=10) |
| --- | --- | --- | --- | --- | --- | --- | --- | --- |
|  | Mean±SEM (pg/ml) | Mean (pg/ml) | *p* value | Significance | Mean±SEM (pg/ml) | Mean±SEM (pg/ml) | Mean±SEM (pg/ml) | Mean±SEM (pg/ml) |
| GM-CSF | 0.1±0.03 | 0.2±0.04 | 0.09 |  | 0.2±0.1 | 0.2±0.10 | 0.2±0.1 | 0.2±0.1 |
| IL-5 | 0.1±0.03 | 0.6±0.1 | <0.001 | *** | 0.6±0.2 | 1.1±0.3 | 0.5±0.5 | 0.5±0.2 |
| IL-8 | 19±5.2 | 348±102 | <0.0001 | **** | 640±244 | 215±58 | 9.7±6.8 | 234±154 |
| IL-10 | 0.0±0.0 | 0.01±0.01 | - |  | 0 | 0.04±0.04 | 0 | 0 |
| CCL2 | 22±2.5 | 239±544 | <0.0001 | **** | 249±59 | 292±97 | 23±1.0 | 252±142 |
| CCL3 | 2.6±0.9 | 143±33 | <0.0001 | **** | 232±75 | 134±42 | 8.0±5.0 | 93±43 |
| CCL4 | 4.0±0.7 | 58±17 | <0.0001 | **** | 100±46 | 62±15 | 2.6±1.1 | 24±8.7 |
| CCL13 | 80±12 | 121±25 | <0.05 | * | 136±51 | 163±58 | 44±15 | 92±29 |
| Flt-1 | 1198±226 | 1514±154 | 0.06 |  | 1841±224 | 1785±439 | 898±147 | 1122±175 |
| VEGF | 7.2±2.0 | 92±25 | <0.0001 | **** | 63±18 | 128±62 | 17±15 | 119±62 |
| VCAM-1 | 5771±867 | 13628±3241 | 0.09 |  | 21279±7515 | 7624±3858 | 8183±6005 | 11648±4830 |
| ICAM-1 | 22563±1298 | 41316±3782 | <0.001 | *** | 51053±6036 | 52728±7862 | 20004±2597 | 27869±3974 |

**Table 3. Fat pad**

Levels of markers in fat pad tissue. Comparing revision versus primary synovial fluid IL-5, IL-8, CCL2, CCL3, CCL4, CCL13, VEGF and VCAM-1 were significantly up-regulated in revision patients (*p*=<0.05, U-test). The remaining four markers were not different. No significant differences were seen comparing different indications for revision surgery (*p*=>0.05). **p* < 0.05, ** *p* < 0.01, *** *p* < 0.001, and **** *p* < 0.0001.

| Marker | Primary (n=28) | All revisions (n=32) | Primary versus Revision TKA | | Osteolysis,  loose components (n=11) | Primary laxity pattern (n=8) | Progression of OA  (PFJ replacement, n=3) | Clinical diagnosis  of fibrosis (n=10) |
| --- | --- | --- | --- | --- | --- | --- | --- | --- |
|  | Mean±SEM (pg/ml) | Mean (pg/ml) | p value | Significance | Mean±SEM (pg/ml) | Mean±SEM (pg/ml) | Mean±SEM (pg/ml) | Mean±SEM (pg/ml) |
| GM-CSF | 0.2±0.06 | 0.2±0.1 | >0.99 |  | 0.2±0.1 | 0.2±0.1 | 0.4±0.4 | 0.1±0.1 |
| IL-5 | 0.3±0.1 | 0.5±0.1 | 0.07 |  | 0.6±0.1 | 0.7±0.2 | 0.4±0.4 | 0.4±0.1 |
| IL-8 | 18±5.6 | 376±113 | <0.0001 | **** | 883±269 | 149±76 | 126±118 | 81±21 |
| IL-10 | 0 | 0.01±0.01 | - |  | 0 | 0.05±0.04 | 0 | 0.01±0.01 |
| CCL2 | 42±7.0 | 138±26 | <0.001 | *** | 198±61 | 127±26 | 44±15 | 111±39 |
| CCL3 | 5.4±1.2 | 155±36 | <0.0001 | **** | 295±69 | 157±83 | 117±112 | 26±7.7 |
| CCL4 | 10±2.4 | 67±17 | <0.01 | ** | 131±37 | 41±15 | 80±75 | 15±4.8 |
| CCL13 | 114±39 | 102±21 | 0.8 |  | 126±53 | 112±28 | 119±89 | 66±12 |
| Flt-1 | 2273±422 | 1897±224 | 0.32 |  | 1778±326 | 2785±768 | 1646±292 | 1520±223 |
| VEGF | 12±2.1 | 38±13 | 0.94 |  | 35±17 | 36±11 | 8.7±5.2 | 51±33 |
| VCAM-1 | 5502±944 | 9220±1851 | 0.09 |  | 12622±4305 | 4102±384 | 4645±2074 | 10323±2928 |
| ICAM-1 | 37609±3587 | 43955±3705 | 0.25 |  | 58640±7112 | 42858±5113 | 44678±12390 | 29770±3615 |

**Table 4. Synovial membrane**

Levels of markers in synovial membrane tissue. IL-8, CCL2, CCL3 and CCL4 were significantly upregulated in synovial membrane tissue in revision versus primary (*p*=<0.05). The remaining eight markers were not different. There was no difference comparing different indications for revision (*p*=>0.05). **p* < 0.05, ** *p* < 0.01, *** *p* < 0.001, and **** *p* < 0.0001
